# Regulation of cardiomyocyte DNA damage and cell death by the type 2A protein phosphatase regulatory protein alpha4

**DOI:** 10.1038/s41598-021-85616-5

**Published:** 2021-03-18

**Authors:** Jonathan Cowan, Michael R. Longman, Andrew K. Snabaitis

**Affiliations:** School of Life Sciences, Pharmacy and Chemistry, Faculty of Science Engineering and Computing, Kingston University, Penrhyn Road, Kingston-upon-Thames, Surrey KT1 2EE UK

**Keywords:** Cell death, Cell signalling, Mechanisms of disease, Post-translational modifications, DNA damage and repair, RNAi, Phosphorylation, Cell biology, Medical research, Cardiovascular biology, DNA, Kinases, Proteins

## Abstract

The type 2A protein phosphatase regulatory protein alpha4 (α4) constitutes an anti-apoptotic protein in non-cardiac tissue, however it’s anti-apoptotic properties in the heart are poorly defined. To this end, we knocked down α4 protein expression (α4 KD) using siRNA in cultured H9c2 cardiomyocytes and confirmed the lack of DNA damage/cell death by TUNEL staining and MTT assay. However, α4 KD did increase the phosphorylation of p53 and ATM/ATR substrates, decreased the expression of poly ADP-ribose polymerase and associated fragments. Expression of anti-apoptotic proteins Bcl-2 and Bcl-xL was reduced, whereas expression of pro-apoptotic BAX protein did not change. Alpha4 KD reduced basal H2AX Ser139 phosphorylation, whereas adenoviral-mediated re-expression of α4 protein following α4 KD, restored basal H2AX phosphorylation at Ser139. The sensitivity of H9c2 cardiomyocytes to doxorubicin-induced DNA damage and cytotoxicity was augmented by α4 KD. Adenoviral-mediated overexpression of α4 protein in ARVM increased PP2AC expression and augmented H2AX Ser139 phosphorylation in response to doxorubicin. Furthermore, pressure overload-induced heart failure was associated with reduced α4 protein expression, increased ATM/ATR protein kinase activity, increased H2AX expression and Ser139 phosphorylation. Hence, this study describes the significance of altered α4 protein expression in the regulation of DNA damage, cardiomyocyte cell death and heart failure.

## Introduction

Cellular alpha4 (α4) was first cloned from B-lymphocytes as an immunoglobulin binding protein^1^ and then later identified as tap42 in yeast^2^. Several studies have shown α4 associates with all three type 2A protein phosphatase (T2APP) catalytic subunits in a non-catalytic manner^3–7^, which has been reported to be inhibitory^5,8,9^. However, a caveat for this inhibitory nature may depend on the identity of the substrate^10^. Alpha4 plays a central role in maintaining the expression of T2APP catalytic subunits in numerous tissues^9,11^, by controlling the ubiquitination state and levels of PP2AC^9,12,13^. Hence, an additional consequence of the association between α4 and T2APP catalytic subunits, involves the “protection” of catalytic subunits from polyubiquitination and consequent 26S proteasome-mediated degradation^9^. Interruption of this T2APP-α4 interaction by the genetic ablation of α4 protein expression, has been shown to indirectly knockdown the expression of all T2APP catalytic subunits and induce apoptotic cell death in a number of cell types^9,14^. Hence, α4 is considered to be an endogenous inhibitor of apoptosis, whose role in apoptotic cell death and heart failure is poorly understood.

Severe decompensated heart failure is characterised by the loss of left ventricular myocardial mass, thereby resulting in failure of pump function. Cell death can occur in an uncontrolled irreversible manner termed necrosis, or by a highly regulated process known as apoptosis, which is characterised by a cell committing to a series of cellular events that ultimately results in cell death. In the normal non-diseased heart the proportion of myocytes undergoing an apoptotic programme is very low (0.001–0.01%) compared to ~ 10 to 100-fold higher (0.08–0.25%) in the failing human heart^15,16^. These observations suggest that although the levels of apoptosis in failing human hearts is low as a percentage of total myocardial mass, this chronic persistent level of apoptosis would contribute to the cumulative loss of myocytes and the development of a decompensated phenotype. Apoptosis plays a significant role in many human cardiovascular diseases ranging from myocardial infarction to end-stage heart failure^17–20^. An observed commonality within the myocardial tissue from infarcted and failing hearts described in these studies is the presence of DNA damage, a known stimulus and hallmark of apoptosis. The ability of any tissue to repair DNA damage relies on the activity of a multitude of proteins that constitute the DNA damage response (DDR), which ultimately preserves genomic stability. In general, the DDR involves the initial recognition of the DNA lesion followed by consequent activation of a signalling cascade to repair the DNA damage, of which there are many types and a double strand break (DSB) is considered the most deleterious in terms of cell viability^21^. Central to the DDR are members of the phosphatidylinositol-3 kinase-related kinase (PIKK) family, which is a trinity of serine/threonine protein kinases comprised of ataxia telangiectasia mutated (ATM), ataxia telangiectasia and Rad3-related (ATR) and the DNA-dependent protein kinase catalytic subunit (DNA-PKcs). ATM and DNA-PKcs facilitate the repair of DSBs, whereas ATR is predominantly involved with resolving single stranded DNA structures at stalled DNA replication forks or resected DSBs^22^. DNA DSBs formed as a consequence of genotoxic stress, are rapidly sensed and capped in the DNA domain of the DSB by the MRE11-RAD50-NBS1 (MRN) complex^23^. The capping of the DSB by the MRN complex facilitates the recruitment and activation of ATM to the DSB^24^. One of the primary events in response to DNA damage is the phosphorylation of the histone H2A variant H2AX at Ser139^25,26^, which is considered a canonical target of the DDR PIKK family of kinases^27–31^. A consequence of H2AX phosphorylation at Ser139 by ATM, is it forms docking sites both proximal and distal to the DSB lesion^29,32^ to facilitate the recruitment of many other proteins that comprise the DDR^33^. Activated ATM also phosphorylates the pro-apoptotic tumour suppressor protein p53 on multiple residues (Ser15^34–36^, Ser20^36^ and Ser46^36^) in response to DNA damage. If the DNA damage is too severe so that genomic stability cannot be restored, the cell is then likely to undergo DNA damage-induced apoptosis and cell death. Apoptosis is in part regulated by a family of B cell chronic lymphocytic leukaemia (BCL)-2 proteins, which is further been categorised into either anti- and pro-apoptotic sub-families of proteins^37^. Severe heart failure is associated with elevated levels of pro-apoptotic BCL-2 associated x (Bax) protein expression^38^ and over expression of the BH4 domain of the anti-apoptotic BCL-2-related gene long isoform (Bcl-x_L_) protein has been shown to avert the onset of heart failure^39^. This evidence suggests that both Bcl-x_L_ and Bax members of the BCL-2 family of proteins may be involved in the pathogenesis of heart failure^40^.

It has been known for many years that anti-cancer anthracycline agents such as doxorubicin can induce cardiotoxicity^41,42^, which has since been confirmed by many studies^43,44^ and is thought to occur via inhibition of topoisomerase 2β^45^. In non-cardiac cells, the overexpression of α4 protein protects against cell death induced by doxorubicin and nutrient withdrawal^9^. However, the regulation of doxorubicin-induced cardiotoxicity by the T2APP-α4 protein signalling axis is undefined.

The DDR is driven by protein phosphorylation and evidence suggests that the T2APPs and α4 may play a modulatory role^9,46,47^. In the current study, we report that the short-term (4 days) knockdown of α4 protein altered the expression and activity of proteins involved in the DDR and apoptosis, whereas sustained longer-term (8 days) knockdown of α4 protein expression induced a significant loss of cell viability and subsequent cell death. Short-term α4 protein knockdown (i) increased the expression of pro-apoptotic Bax and decreased expression of anti-apoptotic Bcl-2 and Bcl-xL proteins and (ii) augmented the sensitivity of cardiomyocytes to doxorubicin-induced DNA damage and reduced cell viability. We also show that the loss of α4 protein downregulates H2AX Ser139 phosphorylation, which was rescued by the subsequent re-expression of α4 protein. In adult cardiomyocytes, the overexpression of α4 protein significantly enhanced the expression of PP2A, but not PP6C, whilst enhancing H2AX Ser139 phosphorylation in response to doxorubicin. End-stage heart failure induced by pressure overload for 9 weeks was associated with (i) decreased α4 protein expression, (ii) increased activity of ATM/ATR protein kinases and (iii) increased H2AX expression and Ser139 phosphorylation. This study illustrates the importance of maintaining α4 protein expression in the failing heart, to offset the induction of DNA damage and cell death by apoptosis.

## Results

### Activation of the DNA damage response (DDR) by α4 protein knockdown

**Figure 1:**
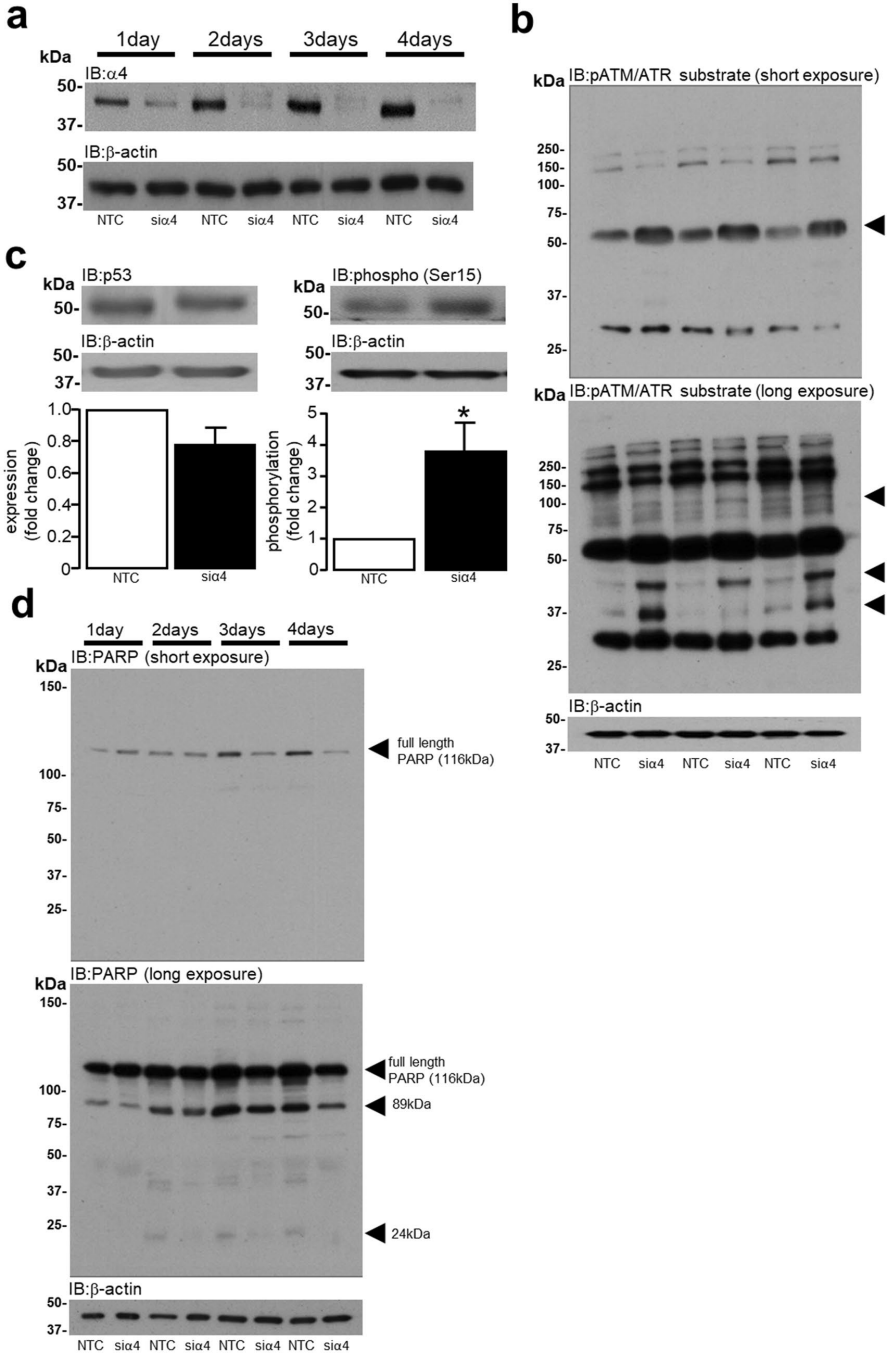
Regulation of the DNA damage response by α4 protein expression in cardiomyocytes. H9c2 cardiomyocytes were transfected with either 50 nM non-targeting control (NTC) siRNA or 50 nM siRNA specific to rat α4 protein (siα4) for 1–4 days (n = 4) and the KD of α4 protein expression was confirmed by Western analysis using an antibody raised against human α4 (**a**). α4 protein expression was knocked down for 4 days and Western analysis (short and long exposures) was used to probe samples (n = 3) with an antibody specific to phosphorylated **S**/**T**QG motifs within ATM/ATR substrates. Arrows indicate substrates whose phosphorylation status (n = 3) was elevated in response to α4 KD (**b**). The expression and phosphorylation (Ser15) of p53 were determined by Western analysis using rodent-specific antibodies raised against mouse p53 protein in samples following 4 days of α4 KD, n = 5 (**c**). The expression of PARP was determined by Western analysis (short and long exposures) following 1–4 days of α4 KD, n = 3. Arrows indicate full length PARP (116 kDa) and cleaved fragments (89 kDa and 24 kDa) (**d**). All data represents data mean values ± SEM and statistical significance vs. non-targeting control (NTC) was determined by a one-tailed Student’s T-test. Expression of β-actin was used to confirm equal protein loading between samples.

The expression of α4 protein was successfully knocked down over a period of 1–4 days by α4 targeted siRNA (Fig. 1a) using protocols previously described^11^. The activity of kinases central to the DDR such as ATM and ATR was determined using an antibody recognising phosphorylated **S**/**T**Q motifs in proteins following 4 days of α4 KD. Figure 1b shows that several proteins underwent enhanced phosphorylation as indicated by arrows, however there were also proteins at a higher molecular weight (~ 150–200 kDa) that appeared to be dephosphorylated. Furthermore Fig. 1c demonstrates that total p53 expression, which can become elevated in response to genotoxic stress^48–50^, remained unchanged despite being significantly (*P = 0.0148) phosphorylated at Ser15 in response to α4 KD. The expression of full length 116 kDa PARP, which is considered to be another protein involved in the DDR and an enzyme stimulated by the presence of single and double strand breaks^51^, was reduced following 4 days of α4 KD (Fig. 1d). Interestingly, the abundance of the cleaved fragments (89 kDa and 24 kDa) was also reduced in response to α4 KD, thereby suggesting that PARP protein was not cleaved in response to α4 KD, but subject to total protein degradation. These data suggest that the knockdown of α4 protein in cardiomyocytes alters kinase activity and the expression of proteins involved in the DDR that is indicative of DNA damage.

### Knockdown of α4 protein expression and cell viability

**Figure 2:**
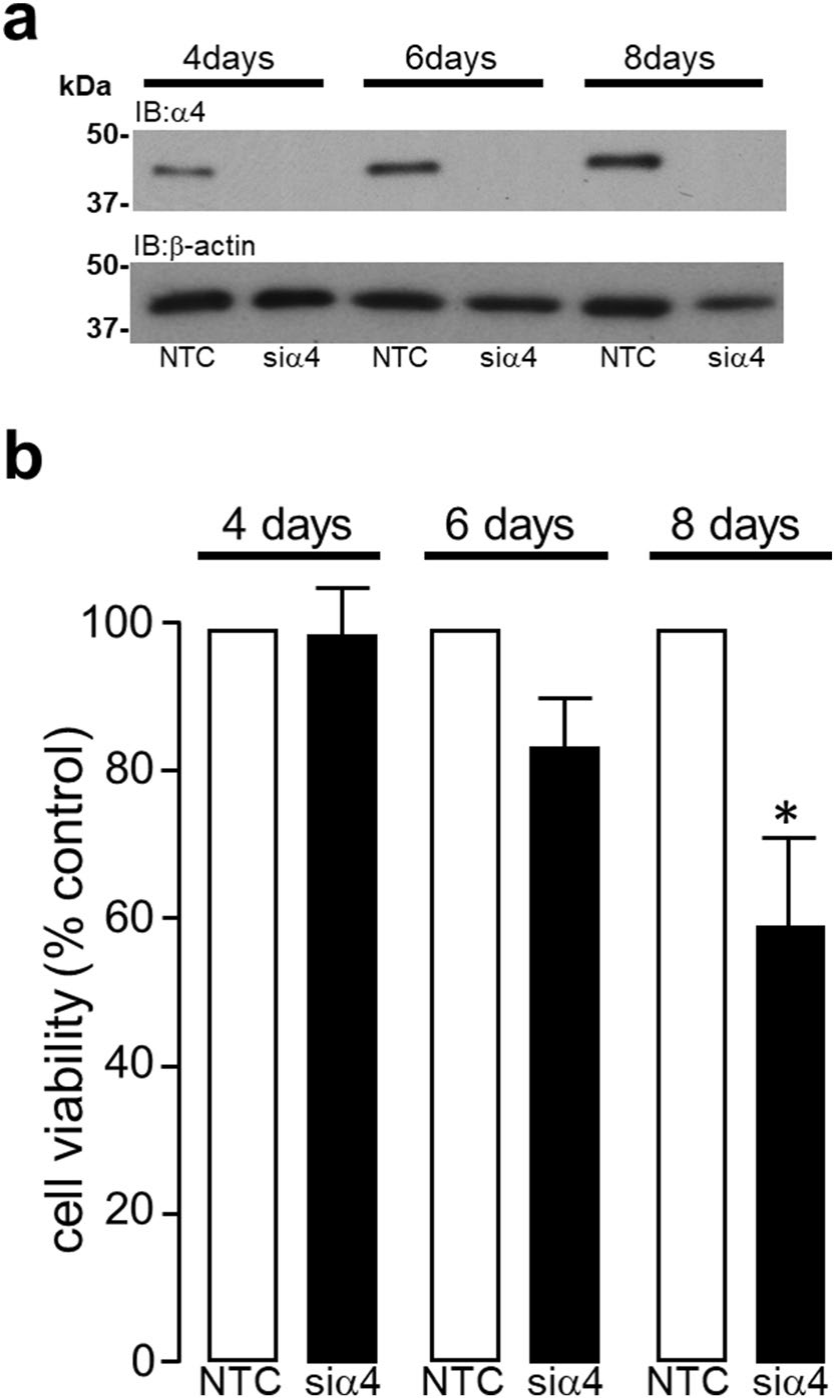
Long-term knockdown of α4 protein expression regulates DNA damage and cell death. H9c2 cardiomyocytes were transfected with either 50 nM non-targeting control (NTC) siRNA or 50 nM siRNA specific to rat α4 protein (siα4) for 4, 6 and 8 days and the KD of α4 protein (n = 4) was confirmed by Western analysis (**a**). The viability of H9c2 cardiomyocytes following the KD of α4 protein expression for 4, 6 and 8 days was determined by MTT assay, data represents mean values ± SEM of 3 individual experiments (**b**). All data represents mean values ± SEM and statistical significance was determined by ANOVA followed by a multiple comparison Tukey’s modified test.

Figure 2a clearly illustrates that α4 protein expression was completely abolished after 4–8 days transfection with siRNA targeted against mRNA specific for rat α4 protein. This was associated with a progressive loss in cell viability, which reached significance (*P < 0.05) at 8 days post-transfection with α4 protein specific siRNA (Fig. 2b). Interestingly, the absence of a pathological effect following 4 days of α4 KD was observed despite our previous evidence demonstrating that the expression of all three T2APPs would have been almost completely abolished at this time point^11^.

### Knockdown of α4 protein expression sensitizes cardiomyocytes to doxorubicin-induced DNA damage and cell death

**Figure 3:**
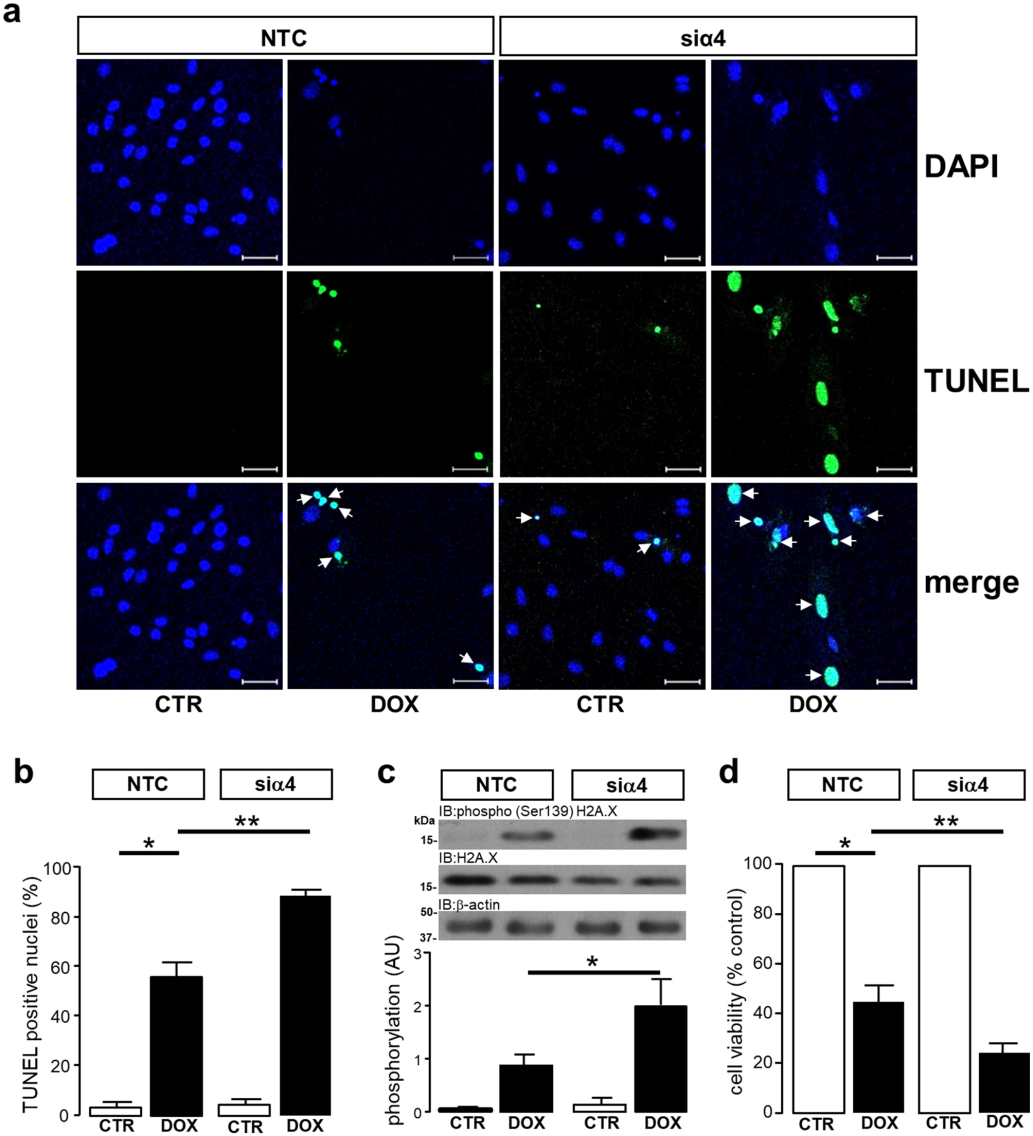
Alpha4 protein regulates doxorubicin-induced DNA damage and cytotoxicity in cardiomyocytes. H9c2 cardiomyocytes were transfected with either 50 nM non-targeting control (NTC) siRNA or 50 nM siRNA specific to rat α4 protein (siα4) for 4 days. H9c2 cardiomyocytes were exposed to either DMEM vehicle control (CTR) or 1 µM doxorubicin (DOX) for 24 h on day 3. DNA fragmentation in H9c2 cardiomyocytes imaged at × 100 magnification was determined by TUNEL assay. Nuclei were stained with DAPI and samples were excited at 405 nm, whereas DNA fragmentation in samples was determined by fluorescein-conjugated TUNEL and excited at 488 nm. White arrows in merged images indicate positive TUNEL stained nuclei, n = 3, scale bar = 50 µm (**a**). DNA damage was quantified by counting DAPI stained nuclei followed by the number of TUNEL positive nuclei in 5 randomly selected regions of interest within the field of view, n = 3 (**b**). H9c2 cardiomyocytes were transfected with either 50 nM non-targeting control (NTC) siRNA or 50 nM siRNA specific to rat α4 protein (siα4) for 4 days. H9c2 cardiomyocytes were exposed to either DMEM vehicle control (CTR) or 100 nM doxorubicin (DOX) for 24 h on day 3. Phosphorylation of H2AX at Ser139 was then determined by Western analysis using a phosphospecific antibody raised against residues surrounding Ser139 of human H2AX, n = 4 (**c**) and cell viability was determined by MTT assay, n = 3 (**d**). All data represents mean values ± SEM and statistical significance was determined by ANOVA followed by a multiple comparison Tukey’s modified test. Expression of β-actin was used to confirm equal protein loading between samples.

Despite the short-term (4 day) knockdown of α4 protein not inducing any significant cell death or DNA damage, it was important to investigate whether α4 KD could modulate the DNA damage in response to genotoxic stress. To this end the topoisomerase 2β inhibitor^45^ doxorubicin was used to induce DNA damage as indexed by the TUNEL assay (Fig. 3a). The quantification of TUNEL positive nuclei in Fig. 3b shows that DOX induced significant (*P < 0.0001) DNA damage which was significantly (**P = 0.001) exacerbated by the knockdown of α4 protein expression (Fig. 3b). Although Ser139 phosphorylation of H2AX was not significantly (*P* = 0.2121) phosphorylated at Ser139 in response to doxorubicin alone, it was significantly (*P = 0.0019) elevated by the knockdown of α4 protein (Fig. 3c). As shown in Fig. 3d, the DNA damage induced by doxorubicin was associated with a concomitant decrease in cell viability (*P < 0.0001) which was exacerbated by the knockdown of α4 protein expression (**P = 0.0098). These data suggest that the absence of α4 protein expression, sensitizes cardiomyocytes to DNA damage induced by the genotoxic agent doxorubicin.

### Modulation of Bcl-2 family protein expression by α4 protein knockdown

**Figure 4:**
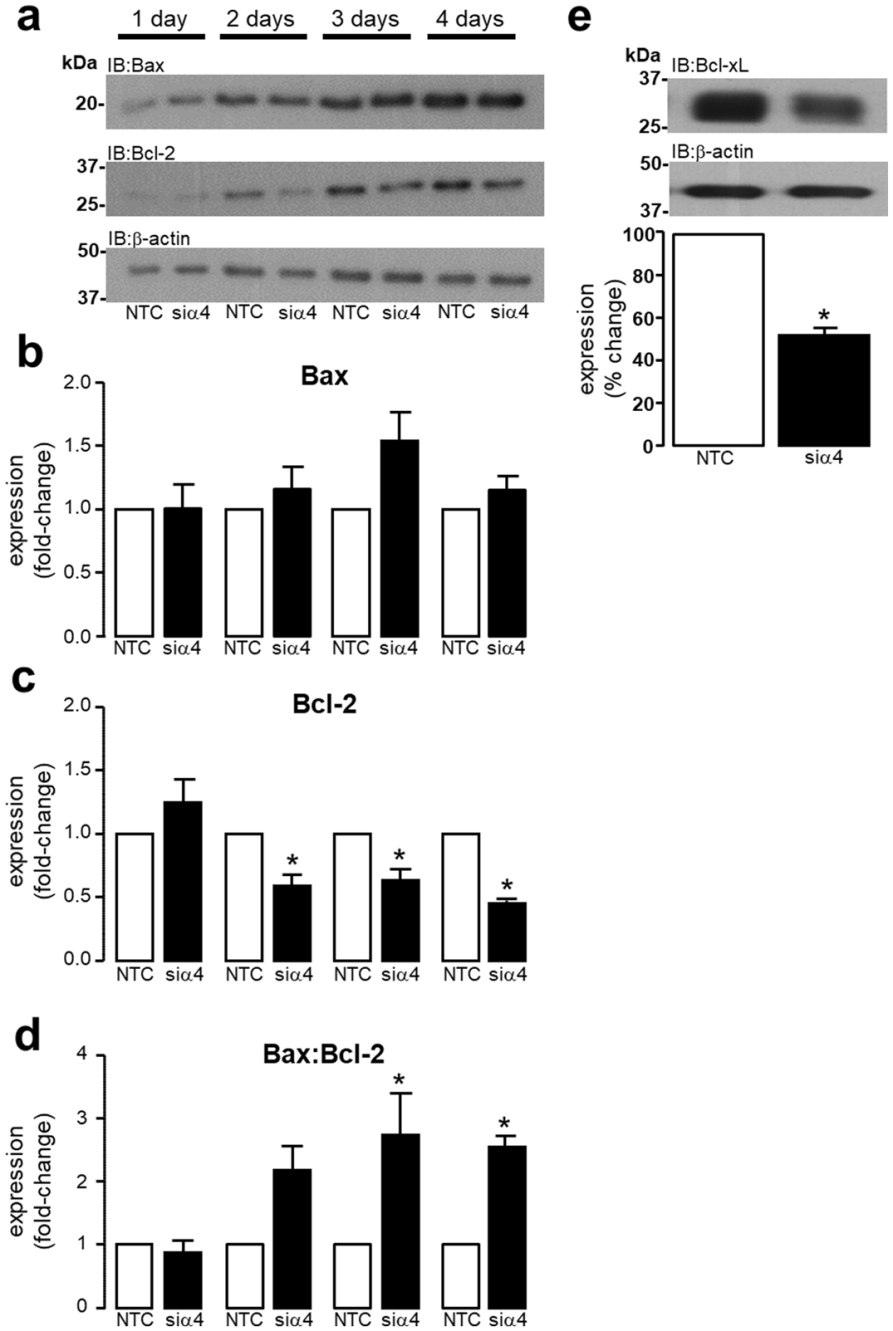
Alpha4 protein knockdown alters expression of BCL-2 family of pro- and anti-apoptotic proteins. H9c2 cardiomyocytes were transfected with either 50 nM non-targeting control (NTC) siRNA or 50 nM siRNA specific to rat α4 protein (siα4) for 1–4 days. The expression of Bax and Bcl-2 protein was determined by Western analysis using antibodies raised against human Bax and Bcl-2 protein (**a**). Quantification of Bax (**b**) and Bcl-2 (**c**) protein expression was determined by densitometry. The Bax:Bcl-2 ratio was calculated by dividing the Bax signal by the Bcl-2 signal (**d**). The expression of Bcl-xL was determined by Western analysis using an antibody raised against human Bcl-xL, in H9c2 cardiomyocytes transfected with either 50 nM non-targeting control (NTC) siRNA or 50 nM siRNA specific to rat α4 protein (siα4) for 4 days (**e**). Where appropriate, statistical significance was determined either by a one-tailed Student’s T-test or by ANOVA followed by a multiple comparison Tukey’s modified test. All data represents mean values ± SEM of 5 individual experiments. Expression of β-actin was used to confirm equal protein loading between samples.

The knockdown of α4 protein expression for 4 days progressively altered the expression of pro-apoptotic Bax and anti-apoptotic Bcl-2 proteins (Fig. 4a). Although our data suggests that there was no significant change in Bax expression following 1–4 days of α4 KD (Fig. 4b), the expression of anti-apoptotic Bcl-2 was significantly decreased following 2 (*P = 0.0162), 3 (*P = 0.0464) and 4 (*P = 0.0007) days of α4 KD (Fig. 4c). Hence, the Bax:Bcl-2 ratio was significantly elevated at 3 (*P = 0.0037) and 4 (*P = 0.0177) days of α4 KD (Fig. 4d). Furthermore, the expression of the anti-apoptotic Bcl-2 family protein Bcl-xL was significantly (*P < 0.0001) reduced following 4 days of α4 KD (Fig. 4e). This suggests that even though there was no significant cell death following 4 days of α4 KD, there were however significant changes in the expression of proteins that can drive the apoptotic process.

### The phosphorylation of H2AX at Ser139 is regulated by α4 protein in cardiomyocytes

**Figure 5:**
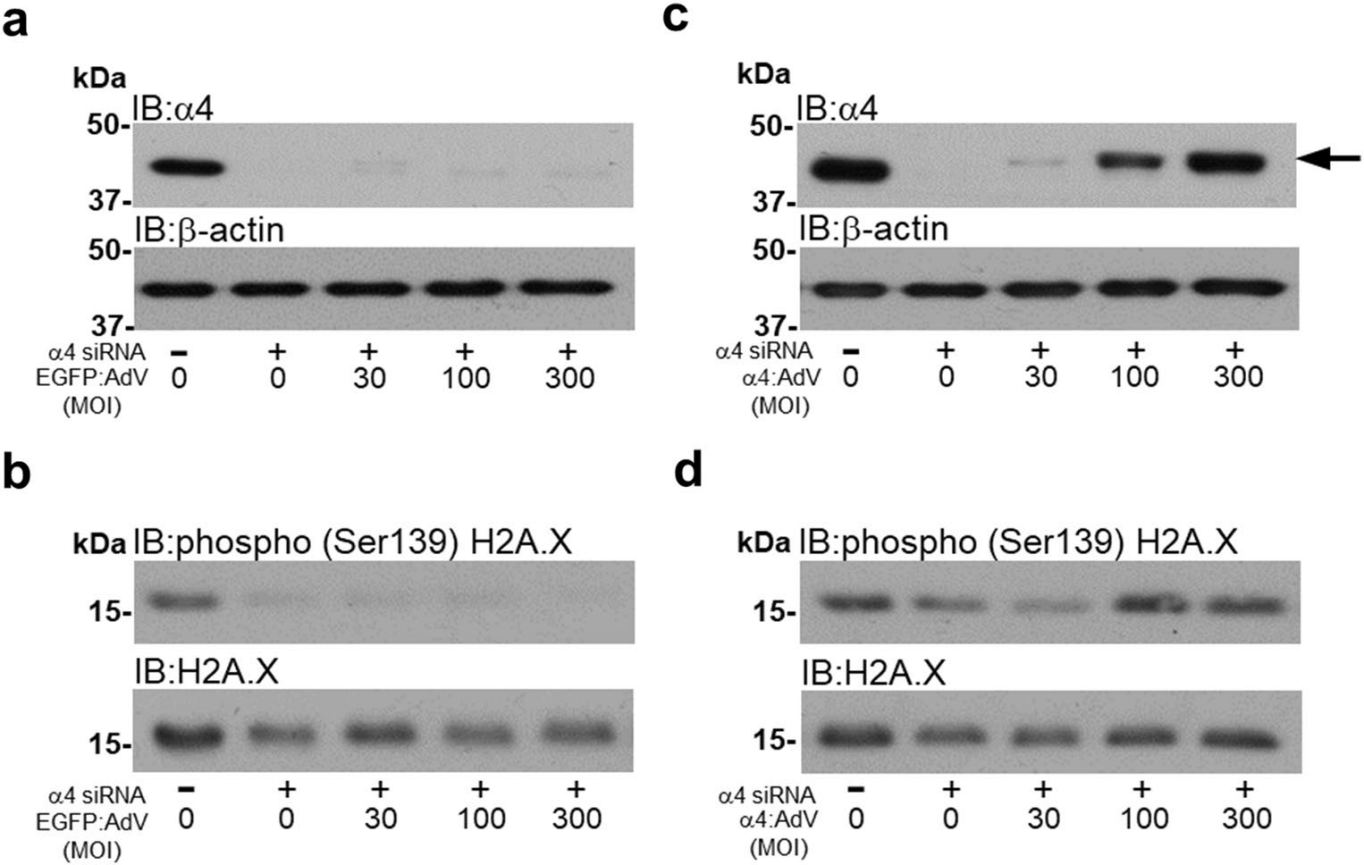
Recovery of α4 protein expression regulates H2AX Ser139 phosphorylation in cardiomyocytes. H9c2 cardiomyocytes were transfected with either 50 nM non-targeting control (NTC) siRNA or 50 nM siRNA specific to rat α4 protein (siα4) for 4 days. On day 3, cardiomyocytes that were infected with either EGFP (**a** and **b**) or α4 (**c** and **d**) expressing adenoviruses at 30, 100 and 300 MOI for 1 h and harvested 24 h later. The expression of α4 and phosphorylation of H2AX at Ser139 was determined by Western analysis using an antibody raised against human α4 and residues surrounding Ser139 of human H2AX, respectively. Western immunoblots are representative of 3 individual experiments. Expression of β-actin and total H2AX was used to confirm equal protein loading between samples.

The phosphorylation status of H2AX at Ser139 has previously been reported to be upregulated by the knockdown of α4 protein expression^9^ and is suggested to be a target for T2APPs in non-cardiac cells^52–54^. The regulation of H2AX Ser139 phosphorylation by the T2APP-α4 signalling axis has not been determined in cardiac cells. To this end we knocked down the expression of α4 protein by siRNA, as previously described, but then re-expressed α4 protein using a novel adenovirus co-expressing EGFP and recombinant V5 -tagged rat α4 protein, under separate CMV promoters. The knockdown of α4 protein expression by siRNA was not altered by the subsequent infection with the control EGFP expressing adenovirus (Fig. 5a). Interestingly, the knockdown of α4 protein expression was associated with a loss of H2AX Ser139 phosphorylation and the subsequent infection with the control EGFP expressing adenovirus did not further alter the Ser139 phosphorylation status of H2AX (Fig. 5b). However, the re-expression of α4 protein to pre-knockdown levels, using the adenovirus expressing V5-tagged rat α4 protein (Fig. 5c) increased the phosphorylation status of H2AX at Ser139 (Fig. 5d). These data suggest that Ser139 phosphorylation of H2AX is regulated by α4 protein expression and appears to be independent of PP2AC expression in cardiomyocytes.

### Alpha4 regulates H2AX phosphorylation in adult rat ventricular myocytes

**Figure 6:**
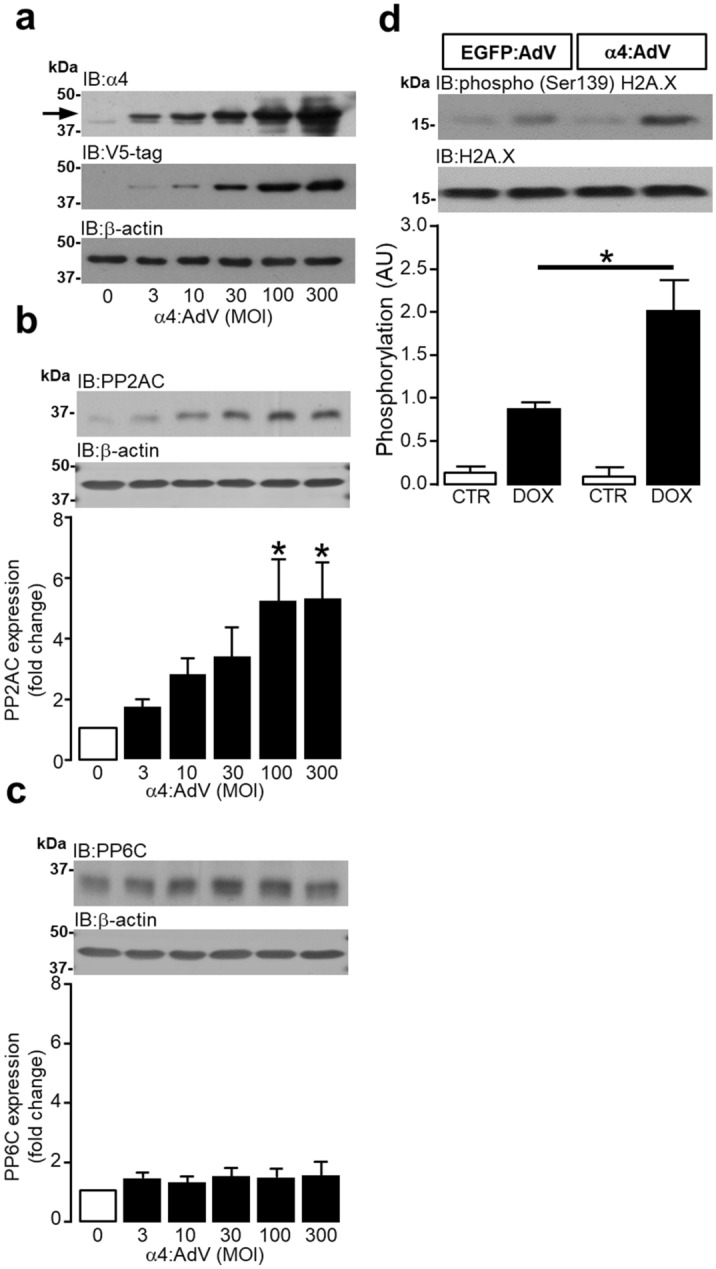
Alpha4 protein overexpression regulates H2AX Ser139 phosphorylation in adult rat ventricular myocytes. Western analysis using antibodies to human α4 protein and V5-tag were used to confirm α4 protein overexpression in ARVM using an adenovirus expressing V5-tagged α4 protein (0–300 MOI). Arrow indicates the expression of the heavier V5-tagged recombinant α4 protein, n = 4 (**a**). The expression of PP2AC (**b**) and PP6C (**c**) in response to adenoviral-mediated α4 protein overexpression was determined by Western analysis using T2APP subunit specific antibodies, n = 5. ARVM were infected with adenoviruses expressing either EGFP or α4 protein (300MOI) and treated with either DMEM vehicle control (CTR) or 100 nM doxorubicin (DOX) for 24 h. Phosphorylation of H2AX Ser139 was determined by Western analysis using an antibody raised against residues surrounding Ser139 of human H2AX, n = 6 (**d**). All data represents mean values ± SEM and where appropriate, statistical significance was determined by ANOVA followed by a multiple comparison Dunnett’s or Tukey’s modified test. Expression of β-actin and total H2AX was used to confirm equal protein loading between samples.

Using protocols previously described^55^, V5-tagged α4 protein was successfully overexpressed in ARVM using an alpha4-expressing adenovirus (Fig. 6a). Using subunit specific antibodies, the overexpression of α4 protein by adenoviral-mediated gene transfer at 100 (*P = 0.0125) and 300 (*P = 0.0114) MOI, significantly increased the expression of the type 2A protein phosphatase catalytic subunit (Fig. 6b) but not the catalytic subunit of PP6 (Fig. 6c). Exposing ARVM infected with the control EGFP expressing adenovirus to doxorubicin, induced a non-significant increase in Ser139 phosphorylation of H2AX. However, infecting ARVM with a α4 expressing adenovirus, did not alter basal H2AX phosphorylation, but did significantly (*P = 0.007) augment the phosphorylation of H2AX in response to doxorubicin (Fig. 6d). Hence, heterologous overexpression α4 protein sensitizes ARVM to DNA damage induced by doxorubicin.

### DNA damage response in end-stage heart failure

**Figure 7:**
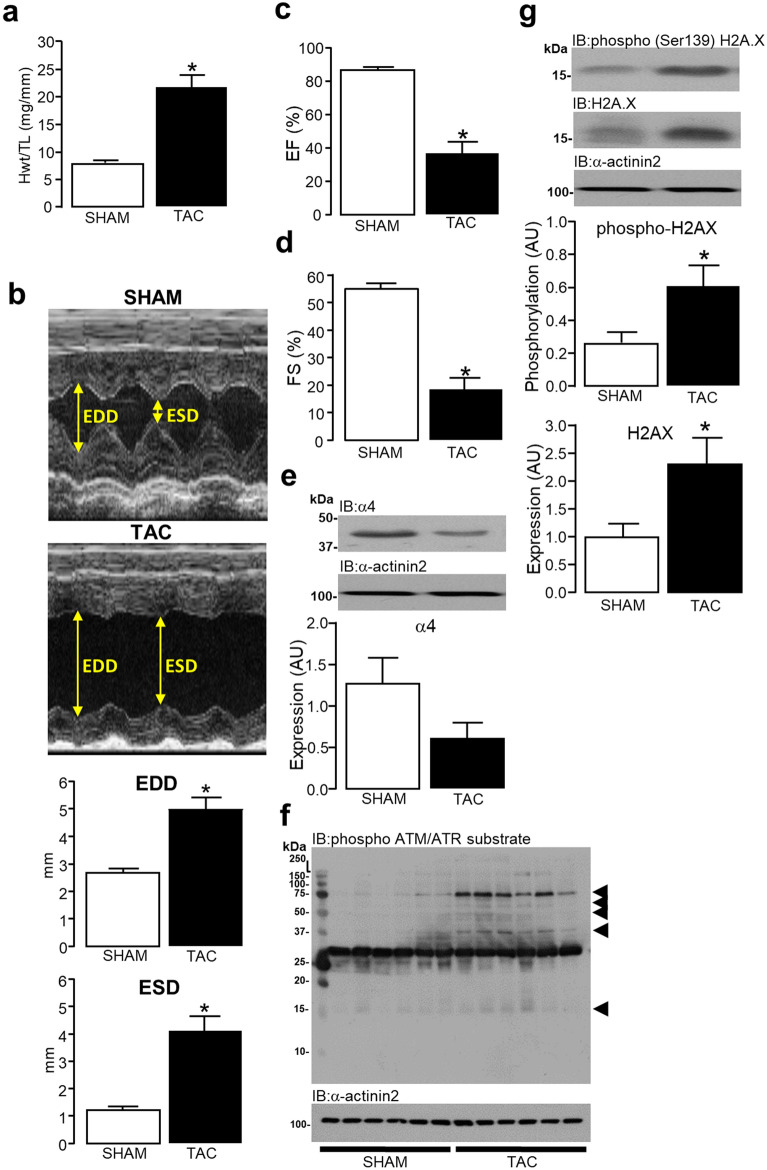
α4 protein expression and DNA damage in pressure overload-induced end stage heart failure. The hypertrophic growth of the heart in response to SHAM or pressure overload induced by transaortic constriction (TAC) for 9 weeks, is expressed as heart weight (mg) normalized to tibia length (mm) (**a**). M-Mode transthoracic echocardiography was used to determine diastolic (EDD) and systolic (ESD) dysfunction (**b**), ejection fraction (**c**) and fractional shortening (**d**) in hearts following 9 weeks of SHAM or TAC surgery. Expression of α4 protein (**e**), phosphorylated ATM/ATR substrates (**f**), H2AX and H2AX Ser139 phosphorylation (**g**) was determined in SHAM and TAC operated hearts by Western analysis using antibodies as described earlier. All data represents mean values ± SEM (6 SHAM/6 TAC) where statistical significance was determined where appropriate by a one or two-tailed Student’s T-test. Expression of α-actinin2 was used to confirm equal protein loading between samples.

Pressure overload-induced by trans-aortic constriction (TAC) for 9 weeks induced a significant (*P = 0.0001) increase (172.4%) in hypertrophic heart growth (Fig. 7a). M-mode transthoracic echocardiography revealed that TAC-operated hearts exhibited significantly compromised left ventricular diastolic (*P = 0.0003) and systolic (*P = 0.0001) function as shown in Fig. 7b, whereas Figs. 7c (*P < 0.0001) and 7d (*P < 0.0001) show significant reductions in both ejection fraction and fractional shortening, respectively. Western analysis of the TAC-operated failing myocardium revealed a non-significant (*P* = 0.1092) > 50% reduction in α4 protein expression compared to hearts from SHAM-operated mice (Fig. 7e). Using an antibody raised against phosphorylated **S**/**T**QG motifs within proteins targeted by the DDR ATM/ATR kinases^56,57^, suggested an increase in ATM/ATR activity and the presence of DNA damage in the failing myocardium (Fig. 7f). Furthermore, levels of H2AX (Ser139) phosphorylation in SHAM and TAC-operated heart tissue was also determined, which was significantly (*P = 0.0367) elevated in hearts from TAC-operated mice. Interestingly, total non-phosphorylated H2AX expression was also determined and found also to be significantly (*P = 0.0299) elevated in failing hearts from TAC-operated mice (Fig. 7g). These observations suggest that pressure overload-induced failing myocardium expresses reduced levels of α4 protein and exhibits significant DNA damage.

## Discussion

The T2APP regulatory protein α4 was reported many years ago to be an endogenous inhibitor of apoptosis in non-cardiac cells^14^ and its role in regulating the expression and activity of T2APPs was described several years later^9^. However, a role for α4 protein in cardiac cells had been poorly defined, until our own recent work reported that α4 protein expression was not only elevated, but its association with PP6C was reduced in hypertrophied cardiac tissue^11^. We and others have previously shown that the deletion of α4 protein expression indirectly decreases the expression of all three T2APPs^9,11^, which in this study appeared to be tolerated for several days by cardiomyocytes, as determined by the absence of cell death. This current study describes the (i) incidence of DNA damage and (ii) the changes that occur in the expression/activity of proteins involved in the DDR and apoptosis during this period of α4 KD, when cell viability appeared to be maintained.

The knockdown of α4 protein expression has previously been shown to increase ATM autophosphorylation in non-cardiac cells^9^. In support, our studies using an antibody raised against the ATM/ATR phosphorylation motif p**S**/**T**QG^56,57^, show that a number of ATM/ATR substrates became phosphorylated, thereby suggesting that ATM/ATR kinase activity was elevated in response to α4 KD. It is worth noting that this antibody may also have detected ATR-phosphorylated substrates. However, evidence suggests that the DDR kinase ATR is primarily recruited to single stranded DNA (ssDNA) at stalled replication forks by the association of its binding partner ATR interacting protein (ATRIP) with ssDNA bound replication protein A (RPA)^22,58^. Hence, it is likely that the phosphorylated substrates detected by the phospho-ATM/ATR substrate antibody, in response to α4 KD, are those substrates predominantly targeted by the DDR kinase ATM. One such substrate is p53, which can be phosphorylated at Ser15 by ATM in response to DNA damage^34–36^. In our study, α4 KD induced significant phosphorylation of p53 at Ser15, thereby implying that α4 KD induced a degree of DNA damage that stimulated the recruitment and activated ATM to the site of DNA damage. Furthermore, α4 KD induced the cleavage of full-length poly(ADP-ribose) polymerase (PARP), an enzyme that participates in the DDR^51^. The cleavage of full length PARP results in the generation of two 89 kDa and 24 kDa fragments that contain the active site and DNA-binding domain of PARP, respectively^59–61^. To our surprise, the cleaved 89 kDa and 24 kDa fragments were also lost, thereby suggesting a wholesale loss of PARP expression. Interestingly, PARP was recently identified as a ubiquitination target for the E3 ubiquitin ligase RNF144A, which is itself activated by DNA damage^62^. This is important to the context of our study as activated RNF144A may ubiquitinate PARP for proteasomal degradation^63^, which may explain the observed disappearance of PARP and it’s fragments in our study.

Alpha4 protein was described many years ago by the Thompson lab as an anti-apoptotic protein ^14^. As in our current study, they rapidly (48–72 h) deleted α4 protein using a variety of techniques, which induced significant cell death in a number of tissues. However, in the present study, there was an absence of DNA fragmentation and cell death, following 4 days of α4 protein expression knockdown by siRNA. However, longer-term knockdown (8 days) of α4 protein expression was associated with significant levels of DNA damage as indexed by terminal deoxynucleotidyl transferase-mediated dUTP nick end labelling (TUNEL) assay and cell death (Supplementary Fig. S1 online). However, knockdown of α4 protein expression for 6 days was shown to induce significant TUNEL staining in the liver of α4-floxed mice injected with a Cre-recombinase expressing adenovirus^14^. It does appear, as though cardiomyocytes may be more tolerant to the absence of α4 protein expression than non-cardiac cell types in which the anti-apoptotic properties of α4 protein have been studied.

Having determined that the knockdown of α4 protein expression induces DNA damage and cell death in cardiomyocytyes, next it was important to determine whether this could exacerbate the effects of a known cytotoxic agent. We have shown, as many other laboratories have done so, that doxorubicin induces significant levels of DNA damage. In addition, we demonstrate that this damage was exacerbated by the knockdown of cellular α4 protein expression, thereby sensitizing cardiomyocytes to not only doxorubicin-induced, but also hydrogen peroxide (H_2_O_2_)-induced (see Supplementary Fig. S2 online) DNA damage. Interestingly, our data shows that the knockdown of α4 protein expression does not itself alter basal H2AX Ser139 phosphorylation (discussed later), but augments H2AX Ser139 phosphorylation and cytotoxicity in response to doxorubicin. This however is only partly supported by previous evidence suggesting that H2AX phosphorylation at Ser139 could be elevated by α4 KD in non-cardiac cells, but prolonged when induced by exposure to either doxorubicin or camptothecin^9^. Hence, our evidence suggests that although the basal phosphorylation status Ser139 of H2AX is not regulated by the T2APP-α4 signalling complex, it does suggest that α4 KD can augment and sensitise cardiomyocytes to DNA damage induced by doxorubicin.

The altered expression levels of BCL-2 family anti-and pro-apoptotic proteins has been used to determine the apoptotic status of cells. The knockdown of α4 protein expression in our current study (i) increased the expression of the pro-apoptotic protein Bax and (ii) decreased the expression of anti-apoptotic Bcl-2, thereby increasing the Bax:Bcl-2 ratio which is suggestive of an increased apoptotic burden within these cells^64^. Furthermore, the expression of the anti-apoptotic protein Bcl-xL was significantly decreased by α4 KD, which has been shown to offset the apoptotic burden when overexpressed in mouse embryonic fibroblasts^14^. The increase in Bax and decrease in Bcl-xL expression is indicative of mitochondrial mediated intrinsic apoptosis, despite unaltered cell viability as determined by the mitochondrial MTT assay. It is possible that there may be a significant lag time between these changes and eventual cell death or that these changes are simply a bystander effect of α4 KD.

Several studies have proposed that Ser139 of H2AX is a T2APP substrate in non-cardiac cells^9,52,53,65^. However, our data earlier in this study suggested that H2AX phosphorylation at Ser139 may not be regulated by the T2APP-α4 complex, which required further investigation. Our previous work has shown that the knockdown of α4 protein using identical protocols for a similar duration as in this current study, significantly downregulated T2APP expression^11^. To this end, we explored this by studying the effects of silencing α4 protein expression using siRNA followed by adenoviral-mediated re-expression of recombinant α4 protein, on H2AX Ser139 phosphorylation. As seen before in this study, α4 KD did not increase but in fact decreased basal H2AX Ser139 phosphorylation and the adenoviral dose-dependent re-expression of recombinant α4 protein, back to pre-silencing levels of expression, restored H2AX Ser139 phosphorylation. Our premise that H2AX Ser139 is not a T2APP target in cardiomyocytes was further supported by our observations in adult ventricular myocytes, where adenoviral-mediated over expression of α4 protein resulted in the increased expression of PP2AC, but not PP6C. Our data suggests that the regulation of PP2AC and PP6C by α4 is different, which is not the first time this has been observed^10^. The observed increase in PP2AC expression in response to α4 protein overexpression is partially supported by evidence reported in HEK293 cells, whereby the heterologous expression of flag-tagged PP2AC was augmented by simultaneous expression of flag-tagged α4 protein^9^. Despite the overexpression of α4 protein significantly elevating PP2AC expression, this did not decrease basal H2AX Ser139 phosphorylation, but actually augmented H2AX Ser139 phosphorylation in response to doxorubicin. Our current data suggests that knockdown or overexpression of α4 protein does alter basal H2AX Ser139 phosphorylation, but that Ser139 of H2AX is not a T2APP target in cardiomyocytes. The augmented H2AX Ser139 phosphorylation in response to doxorubicin, whilst altering α4 protein expression, implies a greater degree of DNA damage in those cells, which may itself be finely controlled by the expression levels of α4 protein.

To put this work into context we also investigated the expression of α4 protein, ATM/ATR kinase activity and phosphorylation of H2AX at Ser139 in hearts subjected to long-term pressure overload. Nine weeks of pressure overload induced a severe failing phenotype that was associated with a > 50% reduction in α4 protein expression. Even though this reduction was not statistically different compared to SHAM animals, it is worth considering that this reduction was a chronic change in α4 protein expression and not an acute response to a short lived stimulus. Hence reduced α4 expression for several weeks may have had deleterious consequences, despite not being statistically different from SHAM operated mouse hearts. A reduction in α4 protein expression in failing hearts was associated with increased myocardial ATM/ATR activity, which is indicative of a stimulated DDR in cardiac tissue subjected to long-term pressure overload-induced DNA damage. These data are not too dissimilar to the data we observed when α4 protein expression was knocked down by siRNA in H9c2 cardiomyocytes earlier in our study. These data highlight the importance of understanding the role of the DDR in heart failure since recent evidence suggests that haplodeficient ATM expression accelerates the development of heart failure following myocardial infarction^66^. In addition, a compromised DDR mediated by the ablation of XRCC1, an enzyme crucial to the repair of DNA single strand breaks, was reported to exacerbate hypertrophic growth and induce heart failure in response to pressure overload^67^. Furthermore, the observed increase in H2AX Ser139 phosphorylation in failing myocardium would be indicative of the DDR, but surprisingly we also observed an increase in total non-phosphorylated H2AX in failing myocardium. This increase in non-phosphorylated H2AX would have offset the observed increase in Ser139 phosphorylation. However, recent evidence suggests that the accumulation of non-phosphorylated H2AX at DNA damage lesions can occur in order to not only stabilise H2AX content at the lesion, but also to expand/amplify the DDR either side of the damaged lesion^68^. Our data confirms that end-stage decompensated heart failure is associated with depressed α4 protein expression and significant DNA damage.

We have shown that the knockdown of α4 protein expression can induce ATM/ATR activation and p53 phosphorylation, thereby suggesting the presence of non-cytotoxic levels of DNA damage and the DDR, but the absence of cell death in cardiomyocytes. However, prolonged α4 protein KD was shown to induce significant DNA damage and eventual cell death. Furthermore, the KD of α4 protein appeared to sensitise cardiomyocytes to DNA damage induced by doxorubicin, which in itself eventually caused significant cell death, possibly through differential expression of BCL-2 anti- and pro-apoptotic factors. We also present two pieces of evidence which suggest that, unlike in non-cardiac cells, Ser139 of H2AX does not constitute a T2APP target. Firstly, the knockdown of α4 protein expression and the subsequent loss of T2APP expression did not increase basal Ser139 phosphorylation and secondly enhanced basal PP2AC expression induced by over expression of α4 protein in adult myocytes, did not reduce basal H2AX Ser139 phosphorylation. Furthermore, our data also suggests that in adult cardiomyocytes, the regulation of PP6C expression by α4 protein is more complex than that of PP2AC. In addition, to contextualise these data, pressure overload induced end-stage decompensated heart failure was associated with reduced α4 protein expression and significant levels of DNA damage and active DDR. We therefore believe that H2AX Ser139 phosphorylation constitutes a novel biomarker of cardiomyocyte DNA damage. We have known for many years that cancer patients undergoing chemotherapy with DNA-damaging genotoxic agents, are predisposed to developing cardiac complications. This current study highlights the importance of understanding how a4 protein may modulate that DNA damage and may allow us to understand how to mitigate any chemotherapy-induced cardiac complications.

## Materials and methods

Animal tissue (adult rat ventricular myocytes) used in this study was obtained in accordance with the UK Home Office Guidance on the Operation of the Animals (Scientific Procedures) Act 1986 (UK), the Directive of the European Parliament (2010/63/EU) and received approval by the local ethics review board at King’s College London. Healthy animals were sacrificed by a schedule one procedure completed by a home office licensed individual such that animal suffering was categorised as minimal. The in vivo animal studies were carried out following guidelines set forth by the American Association for Accreditation of Laboratory Animal Care and the U.S. Public Health Service policy on Humane Care and Use of Laboratory Animals. All mouse studies were approved and supervised by the University of North Carolina at Chapel Hill Institutional Animal Care and Use Committee.

### Materials

#### Antibodies

Sheep polyclonal anti-PP2AC and -PP6C antibodies were a kind gift from Dr BE Wadzinski, University of Vanderbilt and used as previously described^11,69^. Rabbit polyclonal antibody to detect α4 protein (A300-471A) was purchased from Bethyl Laboratories (USA). Antibodies to detect Bax (#2772), Bcl-xL (#2762), phosphospecific ATM/ATR substrate (#6966), IGBP1 (α4) (#5699), PARP (#9542), phosphospecific (Ser139) H2AX (#9718), H2AX (#2595), rodent-specific p53 (#32532), rodent-specific phosphospecific (Ser15) p53 (#12571) and V5-tag (#13202) were obtained from Cell Signaling Technology (USA). The antibody to detect Bcl-2 protein (#610538) was obtained from BD Transduction Laboratories (UK) and the rabbit polyclonal antibody to detect alpha actinin-2 (GTX103219) was purchased from GeneTex (USA). Antibodies to detect β-actin (sc-47778), HRP-conjugated mouse anti-goat (sc-2354) and donkey anti-sheep (sc-2916) secondary antibodies were all purchased from Santa Cruz Biotechnologies (USA). HRP-conjugated donkey/goat anti-rabbit and sheep/horse anti-mouse secondary antibodies were purchased from Cell Signaling Technology (USA).

#### Short interfering RNA

ON-TARGETplus SMARTpool rat specific siRNA to α4 (cat#L-100452-02-0020), ON-TARGETplus non-targeting control (NTC) pool siRNA (cat#D-001810-10-20) and DharmaFECT#1 were purchased from GE Healthcare/Horizon Discovery (Dharmacon).

### Methods

#### H9c2 cardiomyocyte cell culture

H9c2 cardiomyocytes were obtained from ATCC (#CRL-1446) and cultured as described previously^11^. In brief, H9c2 cardiomyocytes cultured for α4 knockdown studies, were initially seeded at ~ 30% confluency and incubated with an appropriate amount of siRNA and maintained in culture for 1–8 days. Doxorubicin or H_2_O_2_ mediated cytotoxicity was determined in H9c2 cardiomyocytes following 4 days of α4 knockdown. H9c2 cardiomyocytes were also re-transfected with siRNA on day 4 when required for longer-term studies requiring α4 knockdown for > 4 days.

#### Isolation and cell culture of adult rat ventricular myocytes (ARVMs)

ARVM were enzymatically isolated from the hearts of adult male Wistar rats (200–250 g, B and K Universal Ltd) as previously described^55^. In brief, ARVM were isolated by collagenase-based enzymatic digestion and then allowed to rest for 2 h post-isolation to form a loose pellet, followed by brief centrifugation at 50 g. Storage medium was then removed and the ARVM pellet was resuspended in sterile cell culture media prior to being cultured in 6-well Nunclon dishes. Freshly isolated ARVM were then infected with the appropriate adenovirus and cultured for a further 24 h as described previously^55^. ARVM were then appropriately treated and processed for Western analysis.

#### Knockdown of protein expression in H9c2 cardiomyocytes by siRNA

H9c2 cardiomyocytes were cultured as described previously^11^. Cultured H9c2 cardiomyocytes were transfected with either 50 nM ON-TARGETplus non-targeting control (NTC) pool siRNA or 50 nM ON-TARGETplus SMARTpool rat α4 specific siRNA (siα4). Transfected H9c2 cardiomyocytes were incubated at 37 °C in 5% CO_2_ for 1 to 8 days. The transfection medium was replaced and cells re-transfected every 4 days when silencing α4 protein expression for > 4 days. The cells were then lysed with 1 × Laemmli sample buffer for subsequent Western analysis.

#### Determination of cell viability by MTT assay

H9c2 cardiomyocytes were seeded at a density of 2 × 10^4^ cells per mL in a 96 well plate and at 30% confluency the cells were transfected with 50 nM non-targeting control (NTC) siRNA or rat α4 specific siRNA using DharmaFECT(#1) transfection reagent. Culture growth media was changed 24 h post-transfection and 3 days after transfection, cells were treated with either 1 µM doxorubicin or DMEM as a vehicle control for 24 h. For the MTT assay, the culture media was replaced with phenol red-free DMEM containing MTT (0.5 mg/mL). The samples were incubated at 37 °C in the dark for 3 h and the media was then removed and replaced with 100µL of DMSO. The plate was then shaken for 15 min, before reading the absorbance at 550 nm using an Infinite M200 PRO plate reader (TECAN, UK). Data was analysed using Magellan data analysis software (Magellan, Taiwan).

#### Recovery of α4 protein expression by adenoviral-mediated gene transfer

Adenoviruses to express EGFP alone or co-express EGFP and α4 protein were purchased from Vector Biolabs (USA). In brief, the adenoviruses expressed either rat specific α4 protein (AdV:α4) containing a C-terminal V5-tag and/or enhanced green fluorescent protein (EGFP) under a separate cytomegalovirus (CMV) promoter. When appropriate, the adenovirus which only expresses EGFP (AdV:EGFP) was used to control for any effects that may have been induced by adenoviral-mediated gene transfer unrelated to the overexpression of α4 protein. The duration of adenoviral infection and calculation of the multiplicity of infection (MOI) was as previously described^55^. For the α4 recovery experiments, α4 protein expression was initially knocked down for 4 days as described in the previous section, except that on the second day H9c2 cardiomyocytes were infected with either EGFP- or α4 protein-expressing adenoviruses at 30, 100 or 300 MOI for 1 h. Cells were then harvested 48 h later for subsequent Western analysis.

#### Pressure overload-induced murine end-stage heart failure

Transaortic constriction (TAC) surgery was completed by Dr Brian Cooley (Core Director, Animal Surgery Core Laboratory) at the McAllister Heart Institute, University of North Carolina, USA. Under ketamine:xylazine anesthesia (100 mg:15 mg/kg, respectively; i.p.) the aortic arch was exposed through an incision made over the suprasternal notch, with muscle and thyroid retraction, and a slight medial incision of the cranial portion of the sternum. A 6–0 silk suture was passed around the aorta between the origin of the innominate and left carotid artery and tied around a 27-gauge blunted needle, with rapid removal of the needle to leave a constricting suture in place around the aorta. The peri-sternal musculature and skin were closed in two layers. Mice were maintained for 9 weeks during which 4 sessions of transthoracic echocardiography were completed at 0 (preoperative), 3, 6 and 9 weeks post-surgery. Mice were then sacrificed by cervical dislocation and hearts rapidly removed and frozen (LN_2_) for subsequent tissue homogenisation.

#### M-mode transthoracic echocardiography

Twelve C57BL/6 male mice (6 SHAM/6 TAC) were pre-trained using restraints on an inclined board (25 degrees) in the supine position (tail down) to allow for conscious acquisition and analysis of M-mode echocardiography. The thoracic area of the mouse was first prepared using a depilation cream and a Doppler was used to image hearts for multiple 5-s intervals with a VisualSonics Vevo2100 ultrasound system. Each echocardiography sessions lasted for no more than 10 min during which time the heart rate was monitored by the ultrasound system. Spatial resolution was 30 µm and the frame rate was 740 fps. Parasternal M-mode images were evaluated measuring left ventricular wall thicknesses and internal diameters at end-systole and end-diastole, with calculation of LV ejection fraction and fractional shortening.

#### Preparation of murine heart tissue homogenates

Whole mouse hearts were homogenised on ice, using a hand held borosilicate glass homogeniser (GPE Scientific) in ice-cold tissue lysis buffer containing; Tris–HCl (20 mM), NaCl (150 mM), Na_2_EDTA (1 mM), EGTA (1 mM), NP-40 (1%), sodium deoxycholate (1%), sodium dodecyl sulphate (0.1%), sodium pyrophosphate (2.5 mM), beta-glycerophosphate (1 mM), Na_3_VO_4_ (1 mM), leupeptin (1 µg/mL) and one mini protease inhibitor cocktail c*O*mplete (Roche Pharmaceuticals, UK), pH 7.5 at a ratio of 100 mg tissue/mL lysis buffer. The tissue homogenate was then centrifuged at 14,000* g* at 4 °C for 30 min to remove any cellular debris and the supernatant removed, to which an appropriate volume of 3 × Laemmli sample buffer was added.

#### Western analysis

Western analysis was carried out as previously described^55,70^. In brief, protein samples were separated by 6% to 15% SDS-PAGE, transferred to PVDF (0.2 µm or 0.45 µm pore size) or nitrocellulose membranes and then probed with primary antibodies. Where appropriate, primary antibodies were detected using donkey anti-rabbit, anti-sheep or anti-goat; goat anti-rabbit; sheep or horse anti-mouse secondary antibodies (1:1000) linked to HRP. Specific protein bands were detected by enhanced chemiluminescence (GE Healthcare, UK) and band intensity was quantified using a calibrated GS-800 densitometer and Quantity One 1-D analysis software v4.6.2 (Bio-Rad, UK). Equal protein loading was determined either by quantifying the content of non-phosphorylated protein (where appropriate), β-actin or alpha actinin-2 within each sample.

#### Determination of DNA damage by confocal microscopy

DNA damage was assessed by terminal deoxynucleotidyl transferase (TdT)-mediated dUTP nick end labelling (TUNEL) of double strand breaks within DNA using a fluorescein-based *in-situ* cell death detection kit (Roche Pharmaceuticals, UK). In brief, H9c2 cardiomyocytes were cultured onto pre-coated (ibiTreat) 35 mm µ-dishes (Ibidi, UK) and treated appropriately. Cardiomyocytes were then fixed using paraformaldehyde (4%) for 10 min at room temperature and briefly rinsed with phosphate buffered saline (PBS). Cardiomyocytes were permeabilised with PBS + Triton X-100 (0.1%) for 15 min at room temperature (RT) and then briefly rinsed with PBS. Cardiomyocytes were then incubated with PBS containing bovine serum albumin (1%) for 1 h at room temperature (RT). The TUNEL stain was prepared with “label solution” according to the manufacturer’s instructions and 50uL was added per µ-dish and incubated in a humidified incubator with 5% CO_2_/95% air for 1 h at 37 °C. The dishes were then briefly rinsed with PBS at RT to which was added 50 µL of ProLong Diamond Antifade mountant with DAPI (Molecular Probes, UK) and a 19 mm coverslip (Thermo Scientific, UK). Dishes were left to cure overnight at RT in the dark. The acquisition of images was performed by exciting the prepared samples with laser lines 405 nm (DAPI stain) or 488 nm (TUNEL stain) using a Zeiss LSM800 laser scanning confocal microscope. Five randomly chosen regions of interest (ROI) per dish were imaged at a magnification of × 100. The DAPI stained nuclei were first counted in each ROI followed by TUNEL positive nuclei and the percentage of apoptotic nuclei was then calculated. TUNEL and DAPI stained Images were merged to determine the % of TUNEL positive nuclei.

### Statistical analysis

Data are presented as mean ± SEM. Where appropriate, data were subjected to either an unpaired Student’s t-test or ANOVA (GraphPad Prism v8.2) to test for significant differences (*P* < 0.05) between groups and further multiple group statistical comparison was completed using either a Dunnett’s or Tukey’s modified t-test if required.

## Supplementary Information

Supplementary Information

## Data Availability

All data generated or analysed during this study are included in this published article (and its Supplementary Information files).

## References

[1] Inui S (1995). Molecular cloning of a cDNA clone encoding a phosphoprotein component related to the Ig receptor-mediated signal transduction. J. Immunol..

[2] Di Como CJ, Arndt KT (1996). Nutrients, via the Tor proteins, stimulate the association of Tap42 with type 2A phosphatases. Genes Dev..

[3] Chen J, Peterson RT, Schreiber SL (1998). Alpha4 associates with protein phosphatase 2A, 4, and 6. Biochem. Biophys. Res. Commun..

[4] Nanahoshi M (1998). Regulation of protein phosphatase 2A catalytic activity by alpha4 protein and it's yeast homolog tap42. Biochem. Biophys. Res. Commun..

[5] Nanahoshi M (1999). Alpha4 protein as a common regulator of type 2A-related serine/threonine protein phosphatases. FEBS Lett..

[6] Murata K, Wu J, Brautigan DL (1997). B cell receptor-associated protein a4 displays rapamycin-sensitive binding directly to the catalytic subunit of protein phosphatase 2A. Proc. Natl. Acad. Sci. USA.

[7] LeNoue-Newton M, Wadzinski BE, Spiller BW (2016). The three type 2A protein phosphatases, PP2Ac, PP4c and PP6c, are differentially regulated by alpha4. Biochem. Biophys. Res. Commun..

[8] Jacinto E, Guo B, Arndt KT, Schmelzle T, Hall MN (2001). TIP41 interacts with Tap42 and negatively regulates the Tor signaling pathway. Mol. Cell.

[9] Kong M, Ditsworth D, Lindsten T, Thompson CB (2009). alpha4 is an essential regulator of PP2A phosphatase activity. Mol. Cell.

[10] Prickett TD, Brautigan DL (2006). The alpha4 regulatory subunit exerts opposing allosteric effects on protein phosphatases PP6 and PP2A. J. Biol. Chem..

[11] Eleftheriadou O (2017). Expression and regulation of type 2A protein phosphatases and alpha4 signalling in cardiac health and hypertrophy. Basic Res. Cardiol..

[12] McConnell JL (2010). Alpha4 is a ubiquitin-binding protein that regulates protein serine/threonine phosphatase 2A ubiquitination. Biochemistry.

[13] Watkins GR (2012). Monoubiquitination promotes calpain cleavage of the protein phosphatase 2A (PP2A) regulatory subunit alpha4, altering PP2A stability and mictotubule-associated protein phosphorylation. J. Biol. Chem..

[14] Kong M (2004). The PP2A-associated protein alpha4 is an essential inhibitor of apoptosis. Science.

[15] Olivetti G (1997). Apoptosis in the failing human heart. N. Engl. J. Med..

[16] Guerra S (1999). Myocyte death in the failing human heart is gender dependent. Circ. Res..

[17] Olivetti G (1996). Acute myocardial infarction in humans is associated with activation of programmed myocyte cell death in the surviving portion of the heart. J. Mol. Cell Cardiol..

[18] Saraste A (1997). Apoptosis in human acute myocardial infarction. Circulation.

[19] Narula J (1996). Apoptosis in myocytes in end-stage heart failure. N. Engl. J. Med..

[20] Aharinejad S (2008). Programmed cell death in idiopathic dilated cardiomyopathy is mediated by suppression of the apoptosis inhibitor apollon. Ann. Thorac. Surg..

[21] Bennett CB, Lewis AL, Baldwin KK, Resnick MA (1993). Lethality induced by a single site specific double-strand break in a dispensable yeast plasmid. Proc. Natl. Acad. Sci. USA.

[22] Weber AM, Ryan AJ (2015). ATM and ATR as therapeutic targets in cancer. Pharmacol. Ther..

[23] Stracker TH, Petrini JHJ (2011). The MRE11 complex: starting from the ends. Nat. Rev. Mol. Cell Biol..

[24] Lee JH, Paull TT (2005). ATM activation by DNA double-strand breaks through the Mre11-Rad50-Nbs1 complex. Science.

[25] Rogakou EP, Nieves-Neira W, Boon C, Pommier Y, Bonner WM (2000). Initiation of DNA fragmentation during apoptosis induces phosphorylation of H2AX histone at serine 139. J. Biol. Chem..

[26] Rogakou EP, Pilch DR, Orr AH, Ivanova VS, Bonner WM (1998). DNA double-stranded breaks induce histone H2AX phosphorylation on serine 139. J. Biol. Chem..

[27] Burma S, Chen BPC, Murphy ME, Kurimasa A, Chen DJ (2001). ATM phosphorylates histone H2AX in response to DNA double-strand breaks. J. Biol. Chem..

[28] Friesner JD, Liu B, Culligan K, Britt AB (2005). Ionizing radiation-dependent gamma-H2AX focus formation requires ataxia telangiectasia mutated and ataxia telangiectasia mutated and Rad3-related. Mol. Biol. Cell.

[29] Savic V (2009). Formation of dynamic gamma-H2AX domains along broken DNA strands is distinctly regulated by ATM and MDC1 and dependent upon H2AX densities in chromatin. Mol. Cell.

[30] Stiff T (2004). ATM and DNA-PK function redundantly to phosphorylate H2AX after exposure to ionizing radiation. Cancer Res..

[31] Ward IM, Chen J (2001). Histone H2AX is phosphorylated in an ATR-depedent manner in response to replication stress. J. Biol. Chem..

[32] Scully R, Xie A (2013). Double strand break repair functions of histone H2AX. Mutat. Res..

[33] Paull TT (2000). A critical role for histone H2AX in recruitment of repair factors to nuclear foci after DNA damage. Curr. Biol..

[34] Banin S (1998). Enhanced phosphorylation of p53 by ATM in response to DNA damage. Science.

[35] Canman CE (1998). Activation of the ATM kinase by ionizing radiation and phosphorylation of p53. Science.

[36] Saito S (2002). ATM mediates phosphorylation at multiple p53 sites, including ser46, in response to ionizing radiation. J. Biol. Chem..

[37] Chipuk JE, Moldoveanu T, Llambi F, Parsons MJ, Green DR (2010). The BCL-2 family reunion. Mol. Cell.

[38] Latif N (2000). Upregulation of the Bcl-2 family of proteins in end stage heart failure. J. Am. Coll. Cardiol..

[39] Sugioka R (2003). BH4-domain peptide from Bcl-xL exerts anti-apoptotic activity in vivo. Oncogene.

[40] Gustafsson AB, Gottlieb RA (2007). Bcl-2 family members and apoptosis, taken to heart. Am. J. Physiol. Cell Physiol..

[41] Singal PK, Iliskovic N (1998). Doxorubicin-induced cardiomyopathy. N. Engl. J. Med..

[42] Lefrak EA, Pitha J, Rosenheim S, Gottlieb JA (1973). A clinicopathological analysis of adriamycin cardiotoxicity. Cancer.

[43] Levis BE, Binlkey PF, Shapiro CL (2017). Cardiotoxic effects of anthracycline-based therapy: what is the evidence and what are the potential harms?. Lancet.

[44] Henriksen PA (2018). Anthracycline cardiotoxicity: an update on mechanisms, monitoring and prevention. Heart.

[45] Zhang S (2012). Identification of the molecular basis of doxorubicin-induced cardiotoxicity. Nat. Med..

[46] Palii SS, Cui Y, Innes CL, Paules RS (2013). Dissecting cellular responses to irradiation via targeted disruptions of the ATM-CHK1-PP2A circuit. Cell Cycle.

[47] Ferrari E (2017). PP2A controls genome integrity by integrating nutrient-sensing and metabolic pathways with the DNA damage response. Mol. Cell.

[48] Thompson T (2004). Phosphorylation of p53 on key serines is dispensible for transcriptional activation and apoptosis. J. Biol. Chem..

[49] Dohoney KM (2004). Phosphorylation of p53 at serine 37 is important for transcriptional activity and regulation in response to DNA damage. Oncogene.

[50] Batchelor E, Mock CS, Bhan I, Loewer A, Lahav G (2008). Recruitment initiation: a mechanism for triggering p53 pulses in response to DNA damage. Mol. Cell.

[51] Bouchard VJ, Rouleau M, Poirier GG (2003). PARP-1, a determinant of cell survival in response to DNA damage. Exp. Hematol..

[52] Rosales KR (2015). TIPRL inhibits protein phosphatase 4 activity and promotes H2AX phosphorylation in the DNA damage response. PLoS ONE.

[53] Chowdhury D (2005). gamma-H2AX dephosphorylation by protein phosphatase 2A facilitates DNA double-strand break repair. Mol. Cell.

[54] Douglas P (2010). Protein phosphatase 6 interacts with the DNA-dependent protein kinase catalytic subunit and dephosphorylates gamma-H2AX. Mol. Cell Biol..

[55] Snabaitis AK, Cuello F, Avkiran M (2008). Protein kinase B/Akt phosphorylates and inhibits the cardiac Na^+^/H^+^ exchanger NHE1. Circ. Res..

[56] Kim S-T, Lim D-S, Canman CE, Kastan MB (1999). Substrate specificities and identification of putative substrates of ATM kinase family members. J. Biol. Chem..

[57] O'Neill T (2000). Utilization of orientated peptide libraries to identify substrate motifs selected by ATM. J. Biol. Chem..

[58] Blackford AN, Jackson SP (2017). ATM, ATR, and DNA-PK: the trinity at the heart of the DNA damage response. Mol. Cell.

[59] Kaufmann SH, Desnoyers S, Ottaviano Y, Davidson NE, Poirier GG (1993). Specific proteolytic cleavage of poly(ADP-ribose) polymerase: an early marker of chemotherapy-induced apoptosis. Cancer Res..

[60] Tewari M (1995). Yama/CPP32 beta, a mammalian homolog of CED-3, is a CrmA-inhibitable protease that cleaves the death substrate poly(ADP-ribose) polymerase. Cell.

[61] Nicholson DW (1995). Identification and inhibition of the ICE/CED-3 protease necessary for mammalian apoptosis. Nature.

[62] Ho SR, Mahanic CS, Lee YJ, Lin WC (2014). RNF144A, an E3 ubiquitin ligase for DNA-PKcs, promotes apoptosis during DNA damage. Proc. Natl. Acad. Sci. USA.

[63] Zhang Y, Liao X-H, Xie H-Y, Shao Z-M, Li D-Q (2017). RBR-type E3 ubiquitin ligase RNF144A targets PARP1 for ubiquitin-dependent degradation and regulates PARP inhibitor sensitivity in breast cancer cells. Oncotarget.

[64] Oltvai ZN, Milliman CL, Korsmeyer SJ (1993). Bcl-2 heterodimerizes in vivo with a conserved homolog, Bax, that accelerates programmed cell death. Cell.

[65] Zhong J (2011). Protein phosphatase PP6 is required for homology-directed repair of DNA double-strand breaks. Cell Cycle.

[66] Jia L (2017). Haplodeficiency of ataxia telangiectasia mutated accelerates heart failure after myocardial function. JAHA.

[67] Higo T (2017). DNA single-strand break-induced DNA damage response causes heart failure. Nat. Commun..

[68] Atsumi Y (2015). ATM and SIRT6/SNF2H mediate transient H2AX stabilization when DSBs form by blocking HUWE1 to allow efficient gammaH2AX foci formation. Cell. Rep..

[69] Kloeker S (2003). Parallel purification of three catalytic subunits of the protein serine/threonine phosphatase 2A family (PP2Ac, PP4c, and PP6c) and analysis of the interaction of PP2Ac with alpha4 protein. Protein Expres. Purif..

[70] Snabaitis AK, D'Mello R, Dashnyam S, Avkiran M (2006). A novel role for protein phosphatase 2A in receptor-mediated regulation of the cardiac sarcolemmal Na^+^/H^+^ exchanger NHE1. J. Biol. Chem..

