# Supplementary material for: Regulation of cardiomyocyte DNA damage and cell death by the type 2A protein phosphatase regulatory protein alpha4
Source: Sci Rep. 2021 Mar 18;11:6293. doi: 10.1038/s41598-021-85616-5 (PMC7973735; doi:10.1038/s41598-021-85616-5)
Supplement: Supplementary file 1 — Supplementary Information [file 41598_2021_85616_MOESM1_ESM.pdf]

Regulation of cardiomyocyte DNA damage and cell death by the type 2A protein phosphatase regulatory protein alpha4

Jonathan Cowan, Michael R Longman and Andrew K Snabaitis.

School of Life Sciences, Pharmacy and Chemistry, Faculty of Science Engineering and Computing, Kingston University, Penrhyn Road, Kingston-upon-Thames, Surrey, KT1 2EE, UK.

Supplementary Figure S1. Alpha4 protein regulates DNA damage in cardiomyocytes. H9c2 cardiomyocytes were transfected with either 50nM non-targeting control (NTC) siRNA or 50nM siRNA specific to rat α4 protein (siα4) for 4, 6 or 8 days. DNA fragmentation in H9c2 cardiomyocytes imaged at x100 magnification was determined by TUNEL assay. Nuclei were stained with DAPI and samples were excited at 405nm, whereas DNA fragmentation in samples was determined by fluorescein-conjugated TUNEL and excited at 488nm. White arrows in merged images indicate positive TUNEL stained nuclei, scale bar = 50μm (**a**). DNA damage was quantified by counting DAPI stained nuclei followed by the number of TUNEL positive nuclei in 5 randomly selected regions of interest within the field of view.

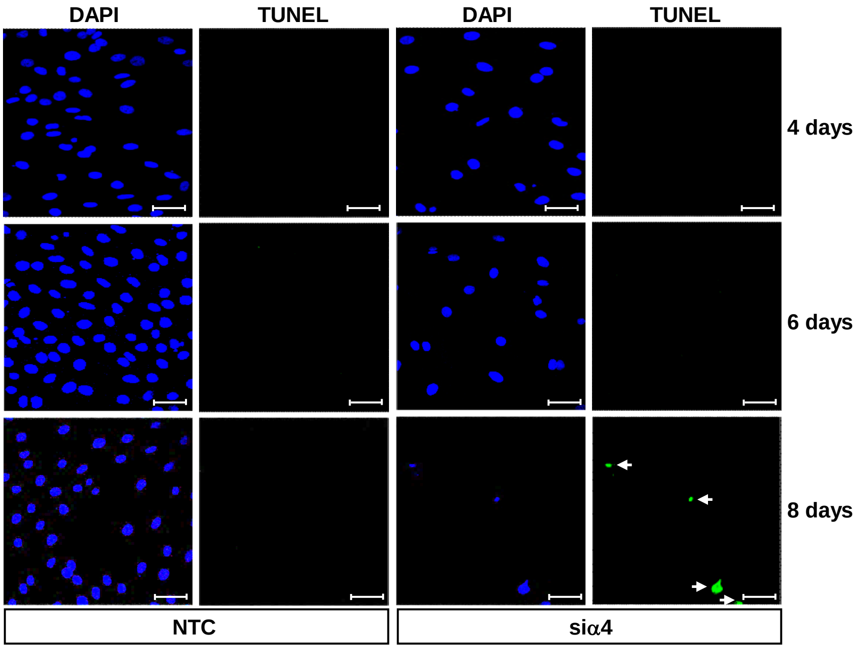

Supplementary Figure S2. Alpha4 protein regulates H_2O_2-induced DNA damage in cardiomyocytes. H9c2 cardiomyocytes were transfected with either 50nM non-targeting control (NTC) siRNA or 50nM siRNA specific to rat α4 protein (siα4) for 4 days. H9c2 cardiomyocytes were exposed to either DMEM vehicle control (CTR) or H_2O_2 (300 μM) for 24 hours on day 3. DNA fragmentation in H9c2 cardiomyocytes imaged at x100 magnification was determined by TUNEL assay. Nuclei were stained with DAPI and samples were excited at 405nm, whereas DNA fragmentation in samples was determined by fluorescein-conjugated TUNEL and excited at 488nm. White arrows in merged images indicate positive TUNEL stained nuclei, scale bar = 50μm (**a**). DNA damage was quantified by counting DAPI stained nuclei followed by the number of TUNEL positive nuclei in 5 randomly selected regions of interest within the field of view (**b**). All data represents mean values ± SEM of 3 individual experiments and **P*<0.05 was determined by ANOVA followed by a multiple comparison Tukey's modified test.

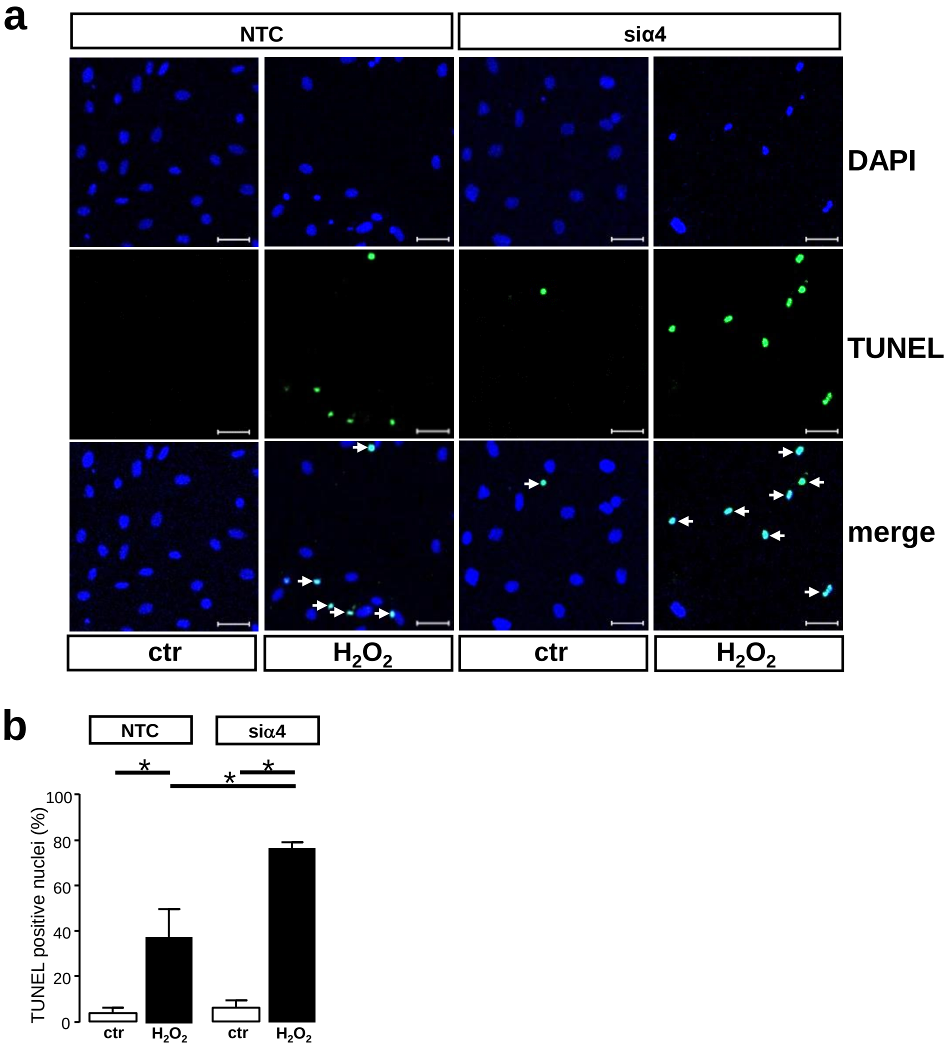

Figure 1

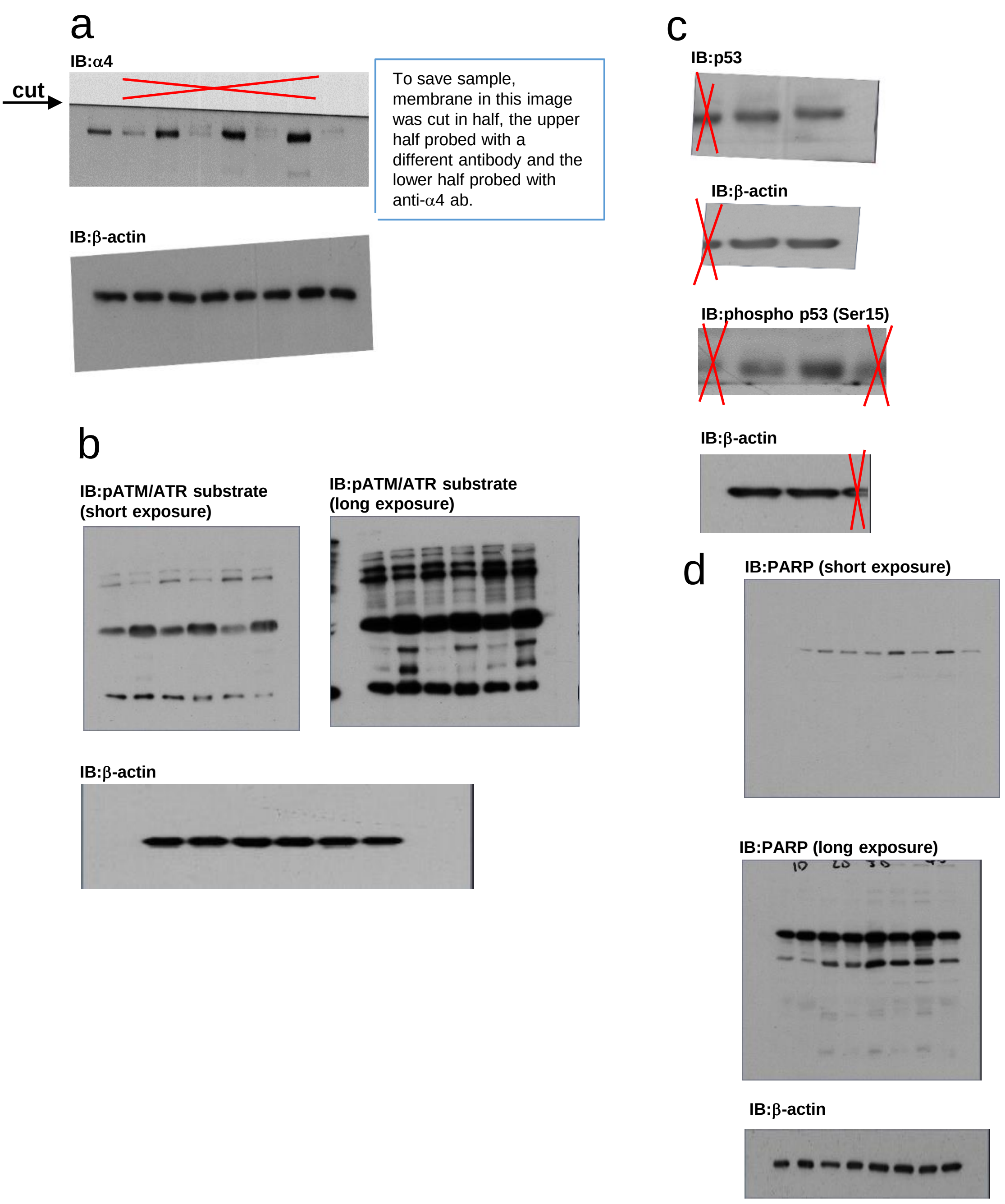
a
IB:α4
cut
To save sample, membrane in this image was cut in half, the upper half probed with a different antibody and the lower half probed with anti-α4 ab.
IB:β-actin
b
IB:pATM/ATR substrate (short exposure)
IB:pATM/ATR substrate (long exposure)
IB:β-actin
c
IB:p53
IB:β-actin
IB:phospho p53 (Ser15)
IB:β-actin
d
IB:PARP (short exposure)
IB:PARP (long exposure)
IB:β-actin

Figure 2

a

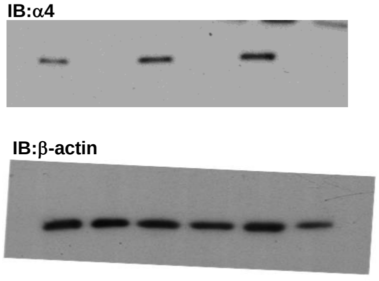

Figure 3

c

IB:phospho (Ser139) H2A.X

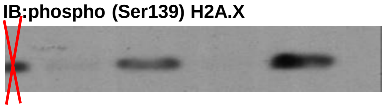

IB:H2A.X

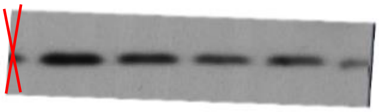

IB:β-actin

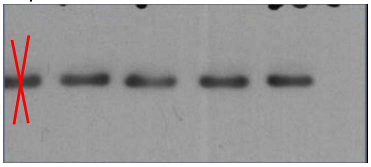

Figure 4

a

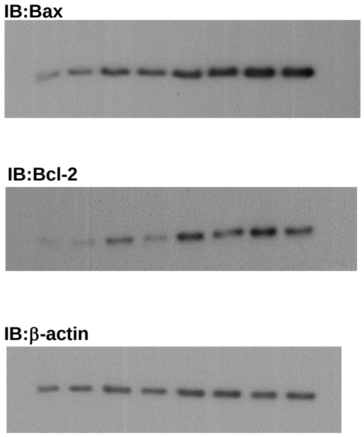

e

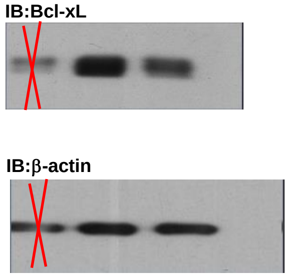

Figure 5

a

IB:α4

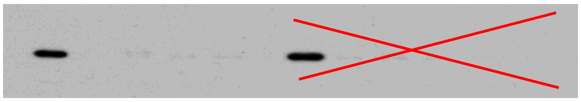

IB:β-actin

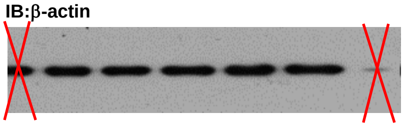

b

IB:phospho (Ser139) H2A.X

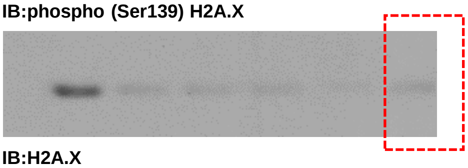

Higher viral dose (1000MOI) omitted from original data in manuscript for clarity

IB:H2A.X

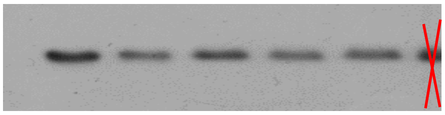

c

IB:α4

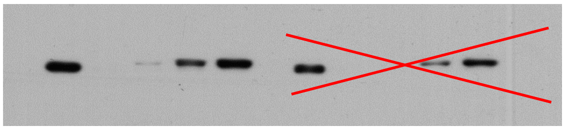

IB:β-actin

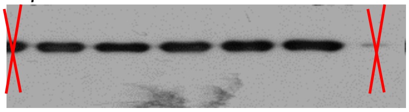

d

IB:phospho (Ser139) H2A.X

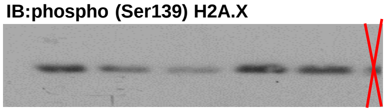

IB:H2A.X

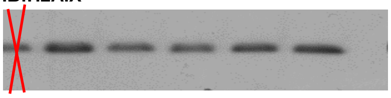

Figure 6

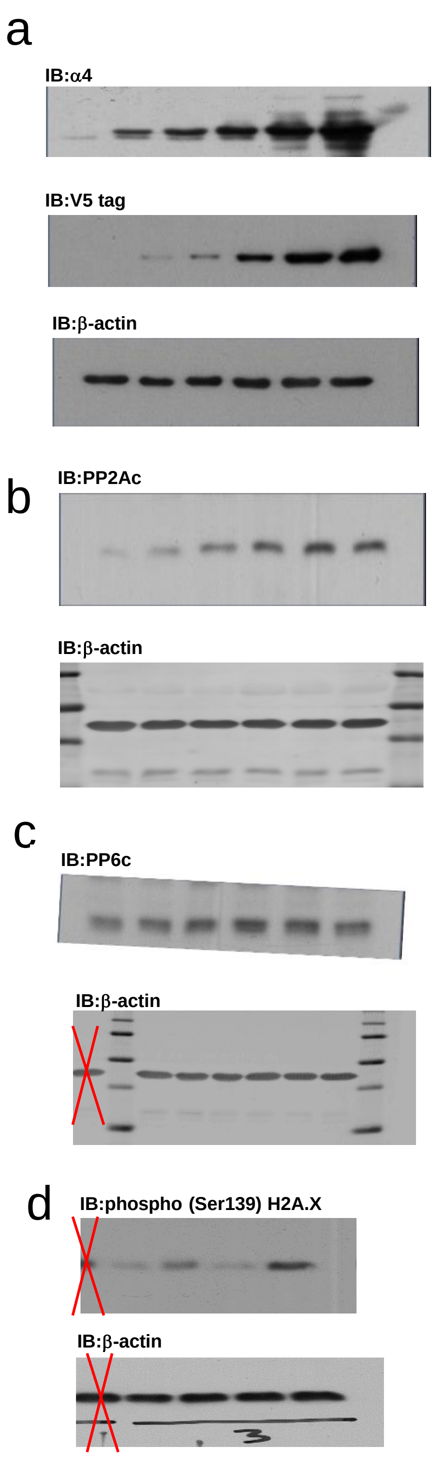
a
IB:α4
IB:V5 tag
IB:β-actin
b
IB:PP2Ac
IB:β-actin
c
IB:PP6c
IB:β-actin
d
IB:phospho (Ser139) H2A.X
IB:β-actin

Figure 7

e

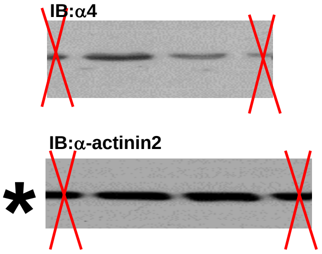

f

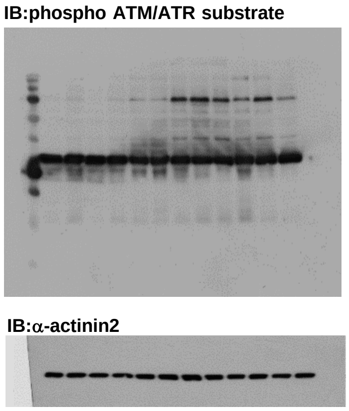

g

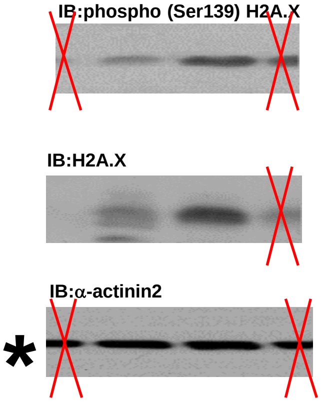

*To conserve heart failure samples, blots in panels **e** and **g** were generated from the same sample and therefore share the same loading control α-actinin2 immunoblot
